# The impact of family factors and communication on recreational sedentary screen time among primary school-aged children: a cross-sectional study

**DOI:** 10.1186/s12889-024-19128-y

**Published:** 2024-06-28

**Authors:** Xueting Ding, Ying Ji, Yuan Dong, Zhijing Li, Yan Zhang

**Affiliations:** 1 Department of Health, Society, and Behavior, Program in Public Health, Susan and Henry Samueli College of Health Sciences, University of California, Irvine, UCI Health Sciences Complex, 856 Health Sciences Quad, 92697 Irvine, CA USA; 2 Department of Social Medicine and Health Education, School of Public Health, Peking University, No.38, Xueyuan Road, Haidian District, 100191 Beijing, People’s Republic of China; 3 Beijing Centers for Diseases Control and Prevention, He Ping Li Zhong Street No. 16, Dong Cheng District,, 100013 Beijing, People’s Republic of China

**Keywords:** Children, Screen time, Sedentary behavior, Family factor, Family communication

## Abstract

**Background:**

Childhood obesity is increasingly recognized as a major public health challenge worldwide, and excessive sedentary screen time is emerging as a key risk factor. This study aimed to assess the recreational screen sedentary time of Chinese primary school-aged children and investigate the relationship between screen-related family factors and the outcome variable.

**Methods:**

Our study used data from a cross-sectional survey collected from fifth-grade students and their parents in Beijing, China, from April to May 2018 (*n* = 2,373). The questions included basic demographic information, family socioeconomic status, students’ and parents’ sedentary and exercising habits, within-family communicational factors, and health belief patterns. The recreational screen sedentary time of the children was compared across demographic groups. The study employed multivariate linear regression models to examine associations between children’s screen time and various family factors, as well as the moderating effect of overall family communication.

**Results:**

Our findings revealed an average daily recreational screen sedentary time of 2.4 h among participants. Screen time significantly varied across demographic categories, including children’s sex, age, residence, parents’ education, household income, family size, and primary family member. After adjustment, the proportion of child-owned digital devices (*p* < 0.01), child’s personal room (*p* < 0.05), family screen-viewing together (*p* < 0.01), and parental screen time (*p* < 0.01) were positively related to children’s recreational sedentary screen time. Parental restrictions on screen time (*p* < 0.001) and attitudes toward reducing sitting time (*p* < 0.01) were correlated with a decrease in children’s screen time. The overall family communication environment significantly moderated the effects of parental practice of restricting children’s screen time (*p* < 0.001), positive reinforcement by parents (*p* < 0.05), and parents’ recreational sedentary screen time (*p* < 0.001).

**Conclusions:**

Our findings underscored the significance of family dynamics, parental practices, and communication in shaping children’s screen time behaviors, providing valuable insights for tailored interventions and strategies to reduce childhood obesity.

**Supplementary Information:**

The online version contains supplementary material available at 10.1186/s12889-024-19128-y.

## Background

As technology advances, electronic devices such as tablets and smartphones have become commonplace, leading to an increased daily amount of time spent on screen-related activities, especially for minors [1, 2]. While certain digital content may be beneficial for cognitive and physical development, screen time has been a major contributor to children’s and teenagers’ sedentary habits, the awake behaviors involving no more than 1.5 metabolic equivalents [3–6]. Ling and Gebremariam [7] recognized childhood obesity as an emerging significant public health concern globally, and excessive sedentary screen time has been identified as a substantial risk factor for weight challenges facing children and adolescents [8–11]. In addition, screen-related sedentary behavior has been associated with a range of children’s physical and psychological burdens, including reduced sleep quality, unhealthy eating habits, increased cardiovascular risk, hindered cognitive growth, impaired self-control, communication skills, emotional challenges, poor eyesight, and decreased bone density [4, 12–17]. At the same time, the WHO recognized “recreational screen time,” defined as the time spent on screens for purposes not related to work or education, as a significant factor in children’s adverse health outcomes [18]. As a result, it would be crucial to study specifically on child recreational screen sedentary time and its predictors.

Parents play a critical role in molding children’s healthy lifestyle behaviors, with the family environment serving as a primary ecosystem supporting their growth and development [7]. Drawing from the ecological model of child development and family systems theory, the family environment — including its physical space, family rules, and interpersonal dynamics within it — constitutes a crucial microsystem [7, 19–23]. The family environment thus may dictate children’s screen-related behaviors. According to social cognitive theory, children adopt behaviors by observing their surroundings, so children’s screen-related behaviors can be significantly influenced by the prevailing related behaviors, perceptions, and attitudes within the family [23, 24]. Prior research has also suggested that routine familial parent–child communications can serve as a mediating factor in how parental influences shape children’s health-related attitudes and behaviors [25–27]. Consequently, family dynamics could be potential predictors of child recreational screen sedentary time, and the overall status of within-family communications may mediate the relationship between various family factors and children’s screen-related behaviors.

With rapid globalization and urbanization, screen time among Chinese children and teenagers has grown dramatically during recent decades, as China has been categorized as an upper-middle-income country [28–30]. While the Physical Activity Guidelines for Chinese recommend limiting daily screen time to under two hours for school-aged minors [31], research has indicated that a significant proportion of Chinese children exceed this recommendation [32]. This overuse has been predominantly in the form of recreational screen sedentary time, which occurs mainly at home and is distinct from active screen time, such as app-based exercise activities. Despite the acknowledged health risks associated with sedentary screen behavior, limited research has specifically focused on assessing the recreational screen sedentary time of Chinese children within their family environments.

In fact, international studies have identified a significant relationship between family factors and children’s sedentary screen time. For example, one study found the number of digital devices at home was related to children’s screen time [33]. Also, parental screen time was shown to be positively associated with children’s screen time [34–38], while parental rules on children’s screen time were associated with reduced screen time among children [34, 35, 37, 39]. In addition, parents’ attitudes, such as concerns about children’s screen time or perceived effects on health, were also found to be related with children’s screen time [37, 40], and higher parental self-efficacy to limit their own screen time has been associated with lower screen time among children [36, 37]. However, there remains a need for a more comprehensive investigation of the diverse family dynamics that may influence screen time among primary school-aged children. Studies considering the diverse family dynamics that may influence children’s screen time, including environmental factors, parental attitudes, and the communicative atmosphere of the family are still needed.

To address the gaps in the current research, this study pursued two primary aims. First, we aimed to assess the recreational screen sedentary time of Chinese primary school-aged children, exploring potential variations across different demographic groups. Second, we aimed to examine whether certain screen-related family factors could be associated with children’s recreational screen sedentary time. Drawing on the literature and theoretical frameworks, we hypothesized that the family’s physical environment, parental attitudes and behaviors, and related parental rules may play pivotal roles in the amount of children’s screen time. A secondary objective of this research is to examine whether overall family communication moderates the link between communicative family factors and children’s screen time. We hypothesized that family communication can act as an effect modifier. Our findings can contribute to family parenting strategies and provide a reference for interventions targeting familial behavioral factors in China and other countries with similar cultural contexts. With the post-COVID transition to more screen-based education and homework, managing children’s screen time has become a complex task for families. This study is pivotal for examining the role of family dynamics in shaping children’s recreational screen usage in this new educational landscape.

## Methods

### Participants

Our study population included primary school students in China, and our sample was collected from fifth-grade elementary schoolers in Beijing from April to May 2018. For each participant, two questionnaires were collected, one completed by the minor and the other by one of their parents. The inclusion criteria were as follows: (1) had activity autonomy to understand and answer the questionnaire correctly; (2) signed the informed consent form; (3) were fifth-grade primary school students who primarily lived at home; and (4) had at least one parent with activity autonomy and signed the informed consent form to complete the parent questionnaire. The exclusion criteria were as follows: (1) did not sign the informed consent form; and (2) primarily lived at school. Additionally, the exclusion criteria during the study period were as follows: (1) requested to withdraw from the study, (2) discontinued due to unexpected events, and (3) indicated that they could not persist in participating in the entire study process.

Considering the personal information of the subjects involved in this study, including family income, education level, and other privacy concerns, we strictly followed the principles of informed consent, voluntary participation, and confidentiality. We fully respected our participants during the data collection process, and they could withdraw from the study at any time if they had any objections. This study was reviewed and approved by the Ethics Committee of the Beijing CDC/Beijing Preventive Medicine Research Center (Ethics Review Approval Number: 201,805).

### Sample size estimation

Initially, our sample size was determined to be 351, calculated based on an estimated prevalence of 32.7% with a margin of error of 0.5 for the estimate derived from random selection. However, considering our adoption of cluster sampling where entire classes were selected as units, we adjusted the required sample size upward to at least 527. This adjustment to 1.5 times the initial size accommodates the cluster sampling method’s design. Since we stratified our sampling process by urban/suburban location and sex, the total sample size was estimated to be at least 2108.

### Sampling

We applied a two-stage cluster sampling method to randomly select four medium-sized average-ranked elementary schools in each of the following areas in Beijing: Haidian district (urban), Chaoyang district (urban), Daxing district (suburban), and Yanqing district (suburban). We then randomly selected 3–4 cohorts of fifth-grade students from each school. A total of 2452 questionnaires were distributed and collected. Excluding the unqualified questionnaires, 2376 valid questionnaires were returned, for a valid response rate of 96.9%. According to our sample size estimation calculation, the sample we obtained for this study met the requirements.

This study utilized a cross-sectional self-administered questionnaire method for students and parents. Students completed their questionnaires under investigator guidance, while parents completed the questionnaires independently. Trained investigators, in collaboration with schools and class teachers, organized on-site surveys. Students submitted their completed questionnaires on-site. The parents completed theirs at home and returned the questionnaires through their children. The classroom teachers reviewed and collected the parent questionnaires uniformly. The questionnaires asked about basic demographics, the family’s socioeconomic status, and the students’ and parents’ sedentary and exercising habits. The questions also included within-family communicational factors as well as daily health belief patterns.

Considering the validity of our questionnaires, we developed our research protocols and survey instruments based on extensive literature review, consulting experts for the validation of the project’s scientific and practical feasibility, and conducting pilot tests to refine the survey based on feedback from experienced field experts. All team members got reviews and training on the survey design, questionnaire, interview guidelines, and data collection techniques to ensure consistent implementation. Preliminary checks of collected survey data are conducted to ensure logical consistency and completeness, with any issues promptly addressed and revised by on-site supervisors. In addition, before data entry and cleaning, each survey was verified for accuracy, and surveys with more than 20% missing data were considered invalid. Data were input into a database using Epidata 3.1 with double data entry verification.

### Measurements

#### Demographic characteristics

The demographic factors of the children included sex (boys or girls), age (10, 11, or 12 years), region of residence (urban or suburban), and ethnicity (minority or not minority). In China, the Han ethnicity constituted the majority (91.6%), while the remaining 55 groups represented various ethnic minorities, such as the Zhuang, Manchu, and Hui [41]. In our study, children of Han Zu ethnicity were categorized as not minority, and those of any other ethnicity were categorized as minority. Family-level demographic factors included variables on the number of children within the family (continuous), main family member (parents, grandparents, others; categorical) who communicated with the participant(s) most, father’s and mother’s education (less than high school, high school, college or more; categorical), and monthly household income (continuous).

#### Family factors

We assessed various variables potentially related with children’s recreational screen sedentary time. Recognizing the role of the family environment as highlighted by the ecological model of child development [19, 20] and family systems theory [22], we included relevant physical environmental factors and parental variables. For physical environmental predictors, the self-reported number of digital devices within the family was included as a continuous variable, and we calculated the proportion of digital devices owned by the children. We also included a variable indicating whether the children had their own room. For parental practices, our analysis included two variables concerning children’s screen time including if the parents restrict their children’s screen time and praise them for less screen time. According to social cognitive theory [24], we included measures of parents’ relevant knowledge, attitudes, and behaviors. Questionnaires on parents’ knowledge of and attitudes toward sedentary screen behaviors were built based on the health belief model [42]. Variable on parents’ related behavior was their daily recreational screen sedentary time. To streamline our analysis, participants’ responses were recategorized. The detailed question items and measures used are outlined in Table 1a.

**Table 1:** a. Family factors

Component	Question items	Measurements
Family objective physical environmental factors	Parent survey: “Which of the following electronic devices do you have in your home? (Multiple choices): (1) Mobile phone (2) Tablet (3) Computer (Desktop, Laptop) (4) Television (5) Game console (PSP, PS2, etc.) (6) Others, please specify.”	Summed number of devices
	Parent survey: “Which of the following electronic devices does your child personally own? (Multiple choices): (1) Mobile phone (2) Tablet (3) Computer (Desktop, Laptop) (4) Television (5) Game console (PSP, PS2, etc.) (6) Others, please specify.”	Summed number of devices and calculated proportion
	Parent survey: “Do you have a separate room for your child? Yes/No”	Yes/No
Family related parental practices	Child survey: “Do your parents restrict your daily screen time? (Including mobile phones, computers, tablets, and all other electronic devices) Strongly agree/Agree/Neutral/Disagree/Strongly disagree”	“Strongly agree” and “Agree” were coded as 1, with all other responses coded as 0.
	Child survey: “Reducing sedentary recreational screen time results in praise from your parents. Strongly agree/Agree/Neutral/Disagree/Strongly disagree”	
	Child survey: “Do your parents spend screen time together with you? Strongly agree/Agree/Neutral/Disagree/Strongly disagree”	
Family associated knowledge, attitudes, and behaviors	Parent survey: “Over the past week, on average how many hours per day do you spend sitting for leisure and entertainment? (This includes freely watching TV, playing on the computer, using a tablet, etc.): a) on a weekday b) on a weekend day	Daily hours (hours/day) calculated: (a x 5 + b x 2) ÷ 7
	Parent survey: “You believe that the longer the sedentary time, the greater the likelihood of becoming overweight or obese. Strongly agree/Agree/Neutral/Disagree/Strongly disagree”	“Strongly agree” and “Agree” were coded as 1, with all other responses coded as 0.
	Parent survey: “You believe that excessive sedentary time is bad for physical health. Strongly agree/Agree/Neutral/Disagree/Strongly disagree”	
	Parent survey: “You personally truly enjoy sitting still and do not like exercising. Strongly agree/Agree/Neutral/Disagree/Strongly disagree”	
	Parent survey: “You believe that you will try to reduce your sedentary time as much as possible. Strongly agree/Agree/Neutral/Disagree/Strongly disagree”	

Furthermore, given the potential role of family communication patterns in affecting children’s behaviors, we developed an indicator to assess the overall status of communication within families. The questions in the questionnaire were designed based on the Family Communication Patterns Scale [43], with adjustments made to fit the cultural context of China. Original responses were scaled from 1 to 5, and we summed the scores to obtain the final communication scale. There are 13 items for our scale, and detailed questions and measures used are outlined in Table 1b. To ensure the reliability of this culturally adapted scale, we conducted an internal consistency analysis using Cronbach’s alpha. We got a Cronbach’s alpha of 0.865 for the raw scores and 0.870 for the standardized scores, indicating a high level of internal consistency.

**Table 1:** b. Family communication

Question items on child survey	Measurements
“I feel very close to my family.” Always/Often/Sometimes/Rarely/Never	Always = 5, Often = 4, Sometimes = 3, Rarely = 2, Never = 1
“I do things together with my family.” Always/Often/Sometimes/Rarely/Never	
“I spend leisure time with my family.” Always/Often/Sometimes/Rarely/Never	
“I avoid my family members at home.” Always/Often/Sometimes/Rarely/Never	Always = 1, Often = 2, Sometimes = 3, Rarely = 4, Never = 5
“I share common hobbies and interests with my family.” Always/Often/Sometimes/Rarely/Never	Always = 5, Often = 4, Sometimes = 3, Rarely = 2, Never = 1
“I actively talk to my family about my feelings.” Always/Often/Sometimes/Rarely/Never	
“When there are conflicts, I keep my thoughts to myself.” Always/Often/Sometimes/Rarely/Never	Always = 1, Often = 2, Sometimes = 3, Rarely = 4, Never = 5
“I argue with my family members.” Always/Often/Sometimes/Rarely/Never	
Have your family members checked your homework? Always/Often/Sometimes/Rarely/Never	Always = 5, Often = 4, Sometimes = 3, Rarely = 2, Never = 1
Do your family members understand your problems and worries? Always/Often/Sometimes/Rarely/Never	
Do your family members know what you do in your free time? Always/Often/Sometimes/Rarely/Never	
Do your family members call you when they are away for a while? Always/Often/Sometimes/Rarely/Never	
Do your family members chat with you? Always/Often/Sometimes/Rarely/Never	

#### Child recreational screen sedentary time and other related variables

The primary outcome of this research was to quantify the average daily recreational sedentary screen time among children. This was assessed through two targeted survey questions designed to capture the duration of weekday and weekend recreational sedentary screen time. The following two specific questions were included in the survey: (1) “During the past 7 days, on school days (weekday), how many hours per day did you typically spend sitting and engaging in leisure activities such as watching television, playing on the computer, using a tablet, etc.?” and (2) “During the past 7 days, on weekends, how many hours per day did you typically spend sitting and engaging in leisure activities such as freely watching television, playing on the computer, using a tablet, etc.?” To calculate the average daily value, we used a weighted formula: weekday screen time was multiplied by five, weekend time was multiplied by two, and the sum of the two variables was then divided by seven to yield a continuous variable representing the average daily recreational sedentary screen time in hours. Of the total 2,376 completed responses, 3 had missing values for the children’s daily recreational sedentary screen time and were excluded from our analysis.

In addition, we captured the two key aspects of children’s time use and included them in our control variables. First, schoolwork-related sedentary time was calculated similarly to the children’s average daily recreational sedentary screen time, using their reported time spent on weekdays and weekends and calculating it as daily average values. It was also a continuous variable in hours. Second, we captured the children’s regular after-school physical activity. The variable was quantified as the number of days per week that participants engaged in at least 30 min of exercise after school. Since no national guidelines regarding extracurricular physical activity were provided in China, the 30-minute threshold was established based on the Healthy China Action guidelines for adults [44].

### Statistical analysis

We ran all of our statistical analyses using SAS 9.4 (SAS Institute, Cary, NC). Descriptive statistics were calculated to provide an overview summary of participants’ characteristics and outcome variable. Categorical variables were summarized using frequencies and percentages, while continuous variables were represented by means and standard deviations. Participants with missing data for any variables were categorized as “missing” group for those specific variables. Then, through bivariate analyses, including t tests and ANOVA, we evaluated significant differences in children’s daily recreational sedentary screen time across demographic groups. We also estimated the means and 95% confidence intervals (CIs) for each group.

A series of multivariate linear regression models were constructed to explore whether children’s daily recreational sedentary screen time was associated with various family factors. Due to distributional concerns from our preliminary tests, the outcome variable underwent log transformation to better adhere to the assumptions of multivariate linear regression analyses. Our adjustment package included the demographic characteristics of the children, their families, and their children’s time spent engaging in other activities. We included both unadjusted and adjusted results for all the models in our analysis. Furthermore, multicollinearity tests were performed for all the independent variables.

With our regression models, we predicted the children’s recreational sedentary screen time based on objective physical environmental factors (Model 1); related parental practices (Model 2); parents’ associated knowledge, attitudes, and behaviors (Model 3); and an integrated model comprising all the aforementioned factors (Model 4). Model 5 was specifically designed to assess the moderating effect of the overall family communication level on the influence of family dynamics. We categorized the participants based on the quartiles of their family communication scale scores, which were calculated by dividing the distribution of collected scores into four equal parts. Each quartile represented a range of scores: the first quartile included scores from the lowest up to the 25th percentile, the second from the 25th to the 50th percentile, the third from the 50th to the 75th percentile, and the fourth included scores from the 75th percentile to the highest. This method aligned with another study using a multi-item scale to evaluate family communication status [45]. We subsequently included this categorical indicator and its interaction with family dynamic variables in Model 4 to assess how different levels of family communication influence the relationship between family dynamics and our outcome of interest. To establish the foundational relationship between family communication and children’s recreational sedentary screen time, before we ran Model 5, we had performed a basic regression analysis assessing the crude association between these two variables.

## Results

The descriptive statistics are shown in Table 2. Of the total 2,373 participants included in our analysis, 53% of them were boys, and 47% were girls. More than half (54%) of the students resided in suburban areas. Most of the students (92%) were not ethnic minorities. Parental education was generally high, with the most common attainment being a high school or associate degree for both fathers and mothers (45% and 43%, respectively). More than 75% of the participants had a monthly household income ranging from 10,000 to 29,999 RMB. More than half (53%) of the participating households had 1 child, and 41% had 2 children. Most of the students (77%) had their parent(s) as the primary family member who predominantly communicated with the children, and 14% of the children communicated mostly with their grandparents. The mean recreational sedentary screen time was 2.4 h (SD = 1.9) for the participants, which exceeded the highest limit of recommended length according to the related guidelines [46].

**Table 2:** Descriptive summary

Variables		*n* (%)
**Gender**	Boys	1258 (53.0%)
	Girls	1115 (47.0%)
**Age of Child**	10	663 (28.5%)
	11	01256 (54.1%)
	12	0404 (17.4%)
**Region: Urban/Suburban**	Suburban	1280 (53.9%)
	Urban	1093 (46.1%)
**Minority**	No	2172 (91.5%)
	Yes	0177 (7.5%)
	Missing	024 (1.0%)
**Father’s Education**	College or Higher	0768 (32.4%)
	High School or Associate	1071 (45.1%)
	Less than High School	0486 (20.5%)
	Missing	048 (2.0%)
**Mother’s Education**	College or Higher	0731 (30.8%)
	High School or Associate	1026 (43.2%)
	Less than High School	0577 (24.3%)
	Missing	039 (1.6%)
**Monthly Household Income in RMB**	< 10,000	893 (37.6%)
	10,000 to 29,999	897 (37.8%)
	>=30,000	398 (16.8%)
	Missing	185 (7.8%)
**Number of Children in the Household**	1	1247 (52.5%)
	2	966 (40.7%)
	3+	153 (6.4%)
	Missing	7 (0.3%)
**Main Family Member Communicated with the Child Most**	Parent(s)	1817 (76.6%)
	Grandparent(s)	321 (13.5%)
	Other	235 (9.9%)
**Child recreational screen sedentary time** ^**1**^		2.4 (1.9)

Note: ^1^This is a continuous variable, so mean (standard deviation) is shown.

According to our bivariate analyses of child recreational sedentary screen sedentary time across various demographic factors (Table 3), boys reported a significantly (*p* < 0.05) greater mean screen time of 2.45 h than girls did at 2.29 h. Our outcome variable also varied significantly (*p* > 0.05) among the age groups of the students. Compared with their urban counterparts, who reported 2.22 h, children in suburban areas spent significantly (*p* < 0.01) more time (2.51 h; 95% CI: 2.41–2.61). There was no significant difference in screen time between children in the minority and nonminority groups. Parents’ education may also contribute to the variance in children’s recreational sedentary screen time (both *p* < 0.0001): Children of parents with less than a high school education recorded the highest screen time, with fathers’ and mothers’ education reflecting similar patterns. Family income also played a significant role in screen time (*p* < 0.01). Children from middle-earning families spent less time on screens, followed by those from higher-income families, and children from families earning less than 10,000 RMB had the highest recreational sedentary screen time, with a mean of 2.57 h. The number of children in the household was significantly (*p* < 0.05) associated with recreational sedentary screen time, indicating that children in larger families might have more screen time. Among the children grouped by major family member, there was a significant variance among the subgroups (*p* < 0.05); the children who communicated with grandparents reported having a maximum amount of recreational sedentary screen time of 2.65 h.

**Table 3:** Comparisons of child recreational screen sedentary time across demographic variables

Variables	Mean (95% Confidence Interval)				*P* value
Gender	Boys		Girls		
	2.4522 (2.3393–2.5650)		2.2895 (2.1902–2.3887)		0.0358*
Age of Child	10	11		12	
	2.5470 (2.3911–2.7028)	2.2961 (2.1955–2.3968)		2.3522 (2.1782–2.5262)	0.0487*
Region: Urban/Suburban	Suburban		Urban		
	2.5102 (2.4054–2.6149)		2.2183 (2.1089–2.3277)		0.0002***
Minority	No		Yes		
	2.3601 (2.2822–2.4379)		2.6131 (2.2741–2.9520)		0.0864
Father’s Education	College or Higher	High School or Associate		Less than High School	
	2.0312 (1.9072–2.1553)	2.5076 (2.3902–2.6250)		2.6135 (2.4475–2.7794)	< 0.0001***
Mother’s Education	College or Higher	High School or Associate		Less than High School	
	2.0256 (1.8967–2.1545)	2.5008 (2.3824–2.6193)		2.6263 (2.4728–2.7797)	< 0.0001***
Monthly Income of Family Member in RMB	>=30,000	10,000 to 29,999		< 10,000	
	2.3184 (2.1243–2.5125)	2.2211 (2.1001–2.3420)		2.5724 (2.4489–2.6959)	0.0009***
Number of Children in the Household	1	2		3+	
	2.3253 (2.2230–2.4276)	2.3817 (2.2630–2.5003)		2.6877 (2.3326–3.0428)	0.0346*
Main Family Member Communicated with the Child Most	Parent(s)	Grandparent(s)		Other	
	2.3248 (2.2392–2.4105)	2.6522 (2.4380–2.8665)		2.3914 (2.2392–2.4105)	0.0161*

Note: Means and 95% confidence intervals of child recreational screen sedentary time are shown across demographic groups; bivariate analyses (t tests and ANOVA) evaluated significant differences in children’s daily recreational sedentary screen time for each group;

* *p* < 0.05, ** *p* < 0.01, *** *p* < 0.001

For our multivariate regression models, all of our models (1 to 5) fit significantly better than the intercept-only model (*p* < 0.0001). No multicollinearity was found among the independent variables in our models (Supplementary Table S1). Several family environmental factors were significantly correlated with children’s recreational screen sedentary time in our study, as shown in Table 4. Most of our significant coefficients demonstrated consistent significance and magnitude with and without adjustment in our analysis (Table 4). See Supplementary Table S2 for further results. In Model 1, an increase in the proportion of devices owned by children within the household was positively associated with greater daily recreational sedentary screen time. Compared with children without their own room, those living in their individual rooms had approximately 7-10% more screen time (unadjusted: coefficient = 0.0970, *p* < 0.001; adjusted: coefficient = 0.0702, *p* < 0.05). The impact of the number of devices at home was not consistent. In Model 2, restricting child screen time was significantly associated with reduced recreational sitting hours. A stricter screen time regimen corresponded to an approximately 11% decrease in children’s recreational sedentary screen time (unadjusted: coefficient=-0.1213, *p* < 0.001; adjusted: coefficient=-0.1112, *p* < 0.001). The practice of praising children for less screen time was associated with a decrease of approximately 5-6% (unadjusted: coefficient=-0.0641, *p* < 0.01; adjusted: coefficient=-0.0542, *p* < 0.05). Conversely, family screen time taken together showed a positive relationship, where an increase in shared screen time was associated with an approximately 9-10% increase in a child’s recreational sedentary screen time (unadjusted: coefficient = 0.0986, *p* < 0.001; adjusted: coefficient = 0.0861, *p* < 0.01).

In Model 3, we found that parents’ recreational sedentary screen time was positively associated with that of their children. For every additional hour spent by a parent in recreational screen sedentary activities, there was a 2% increase in the duration of the child’s same behavior (unadjusted: coefficient = 0.0236, *p* < 0.001; adjusted: coefficient = 0.0207, *p* < 0.001). Moreover, parental attitudes toward reducing their own sitting time were correlated with a reduction in their child’s recreational sedentary screen time of approximately 7% (unadjusted: coefficient=-0.0654, *p* < 0.01; adjusted: coefficient=-0.0715, *p* < 0.01). Model 4 presented a comprehensive approach by integrating various family-associated factors, encompassing physical environment, parental practices, parents’ knowledge, attitudes, and behaviors. The adjusted Model 4 accounted for approximately 8.5% of the variability in our outcome variable. When these elements were analyzed collectively, several factors maintained their significant association with the outcome variable, although with a slightly diminished magnitude of influence (Table 4).

**Table 4:** Multivariate Regression, Coefficients of Model 1–4

Variables	Model 1: Unadjusted	Model 1: Adjusted	Model 2: Unadjusted	Model 2: Adjusted	Model 3: Unadjusted	Model 3: Adjusted	Model 4: Unadjusted	Model 4: Adjusted
*Family objective physical environmental factors*								
Number of devices at home	Not significant	0.0361*					Not significant	Not significant
Proportion of child-owned devices	0.1327***	0.1168**					0.1135**	0.1109**
Child has own room	0.0970***	0.0702*					0.0881***	0.0644*
*Family related parental practices*								
Restrict child screen time			-0.1214 ***	-0.1112 ***			-0.1178 ***	-0.1103 ***
Less screen time gets praise			-0.0641**	-0.0542*			-0.0485*	-0.0378
Family members have screen time together			0.0986***	0.0861**			0.1031***	0.0800**
*Family associated knowledge, attitudes, and behaviors*								
Parent related behavior: recreational screen sedentary time					0.0236***	0.0207 ***	0.0206***	0.0172**
Parent attitude: I will try to reduce my sitting time					-0.0654 **	-0.0715 **	-0.0596 **	-0.0678**

Note: For our Model 1–4, we predicted the children’s recreational sedentary screen time based on objective physical environmental factors (Model 1); related parental practices (Model 2); parents’ associated knowledge, attitudes, and behaviors (Model 3); and an integrated model comprising all the aforementioned factors (Model 4); the outcome variable underwent log transformation to better adhere to the assumptions of multivariate linear regression analyses; adjustment package included gender, age, urban/suburban status, and ethnic minority for the child, father’s education, mother’s education, monthly household income, number of children in the household, main family member communicated with the child most, child schoolwork related sitting time, and number of days per week with 30 + minutes exercising for the child; only significant variables are shown on this table; * *p* < 0.05, ** *p* < 0.01, *** *p* < 0.001

For our last model, firstly, the crude association analysis between family communication quartiles and children’s recreational sedentary screen time revealed that family communication was significantly correlated with our outcome variable (*p* < 0.001). Specifically, as family communication increased from the lowest quartile (reference) to higher ones, we observed a significant increase in leisure-related sedentary screen time. Then, Model 5 validated the role of the overall family communication environment as an effect modifier for the relationship between children’s recreational sedentary screen time and several related family factors. Most of the significant coefficients demonstrated consistent significance and magnitude with and without adjustment (Table 5). The adjusted model accounted for approximately 11.5% of the variability in our outcome variable. For those whose parents restricted their screen time at home, compared to children with low family communication scale scores (1st quartile), those with high scores (4th quartile) had 24.2% less recreational sedentary screen time in hours (unadjusted: coefficient=-0.2822, *p* < 0.001; adjusted: coefficient=-0.2774, *p* < 0.001). Positive reinforcement by parents, such as praising less screen time, was potentially associated with about 9% decrease in our outcome variable (unadjusted: coefficient=-0.1045, *p* < 0.05; adjusted: coefficient=-0.0853, *p* < 0.06). However, the borderline significance in the adjustment model may suggest a less robust association. This potential beneficial effect was largely modified by improved family communication. For children whose parents praised them because of their less time on screens, compared to children with low family communication scale scores (1st quartile), having high scores (4th quartile) was associated with 19.1% more recreational sedentary screen time in hours (unadjusted: coefficient = 0.2072, *p* < 0.01; adjusted: coefficient = 0.1749, *p* < 0.05).

Parents’ related behaviors, particularly their recreational sedentary screen time, were found to be a significant predictor of their children’s behavior. In our analysis, each unit increase in the parents’ reported time was associated with a 5% increase in the children’s time (unadjusted: coefficient = 0.0567, *p* < 0.001; adjusted: coefficient = 0.0526, *p* < 0.001). Improved family communication levels modified this relationship. Among the children with parents who engaged in the same amount of recreational sedentary screen time, those with higher family communication scores (3rd quartile) showed a 5% reduction in their own recreational screen time in hours (unadjusted: coefficient =-0.0493, *p* < 0.01; adjusted: coefficient = -0.0539, *p* < 0.01). This reduction was at 6%, for children in the highest (4th) quartile of family communication scores (unadjusted: coefficient =-0.0605, *p* < 0.001; adjusted: coefficient =-0.0590, *p* < 0.001). See Supplementary Table S3 for further results.

**Table 5:** Multivariate Regression, Coefficients of Model 5

Variables	Model 5: Unadjusted	Model 5: Adjusted
*Family related parental practices*		
Restrict child screen time * Family communication (4th vs. 1st quartile)	-0.2822***	-0.2774***
Less screen time gets praise	-0.1045*	-0.0853^§^
Less screen time gets praise * Family communication (4th vs. 1st quartile)	0.2072**	0.1749*
*Family associated knowledge, attitudes, and behaviors*		
Parent related behavior: recreational screen sedentary time	0.0567***	0.0526***
Parent related behavior: recreational screen sedentary time * Family communication (3rd vs. 1st quartile)	-0.0493**	-0.0539**
Parent related behavior: recreational screen sedentary time * Family communication (4th vs. 1st quartile)	-0.0605***	-0.0590***

Note: Model 5 included objective physical environmental factors, related parental practices, parents’ associated knowledge, attitudes, and behaviors, and interactions of family dynamic variables and quartiles of family communication scale scores; the outcome variable underwent log transformation to better adhere to the assumptions of multivariate linear regression analyses; adjustment package included gender, age, urban/suburban status, and ethnic minority for the child, father’s education, mother’s education, monthly household income, number of children in the household, main family member communicated with the child most, child schoolwork related sitting time, and number of days per week with 30 + minutes exercising for the child; only significant variables are shown on this table; ^§^*p* < 0.06, * *p* < 0.05, ** *p* < 0.01, *** *p* < 0.001

## Discussion

Our study is one of the only studies that specifically targets recreational sedentary screen time among children in China while also comprehensively examining various family environmental factors. On the basis of our cross-sectional survey, which included more than 2000 participants, we found a concerning trend: primary school-aged children in China, on average, were engaged in recreational screen activities for more than two hours daily, with significant variability. These findings echoed similar trends observed in North American studies, where approximately half of school-aged children were reported to spend more than two hours per day on recreational screen use [47, 48]. This average duration exceeded the recommendations of the Physical Activity Guidelines for Chinese individuals, which suggest that school-aged minors spend less than two hours of screen time daily [31]. Similarly, the Canadian Society for Exercise Physiology recommended less than two hours of recreational screen time daily for a “healthy 24 hours” for children aged 5–17 years [49]. Given the possible negative health outcomes associated with excessive screen time, it is crucial to initiate relevant interventions in China to reduce the recreational screen time of children. Additionally, although our study focused only on primary school students, particularly fifth-grade children based on our sample, it provides an instructive direction for future studies focusing on other aged minors in China, for example, Chinese preschool children [18, 31, 46]. 

Our study highlighted the critical influence of family environment elements, such as physical space, parental rules, and interpersonal dynamics, on children’s recreational sedentary screen time. These findings align with our initial hypotheses based on the ecological model of child development and family systems theory. Specifically, unlike another study that identified a correlation between the number of televisions in a home and children’s viewing time in European families [33], our research did not find a significant relationship between the total number of digital devices in a Chinese family and children’s recreational screen time. Instead, we discovered that specific factors such as the proportion of digital devices owned by the child and the availability of a personal room were associated with our outcome variable. These findings suggested that certain elements of children’s immediate physical environment, particularly their personal access to devices and their own space in the household, had a more focused impact on their screen-related behaviors. This was consistent with a similar trend observed in the U.S., where studies have shown a link between a child’s personal access to electronic devices in their bedroom and their overall screen exposure [50, 51]. Additionally, our study revealed a positive correlation between parents watching screens with their children and the length of the children’s screen exposure, which is consistent with findings in the literature [34, 37, 39]. Furthermore, our results showed that parental rules about screen time were associated with the length of children’s screen exposure, as shown consistently in previous studies [2, 52, 53, 54]. Moreover, in line with international studies [55–59], our findings confirmed the significant relationship between parents’ and children’s screen exposure hours, where increased screen time by parents was associated with longer recreational screen use among their children.

Additionally, while previous studies highlighted the mediating role of family communication in shaping children’s health-related behaviors [25–27], our study expanded this understanding to include screen-related behaviors. We found that the overall family communication environment could significantly modify the impact of various family factors on children’s recreational sedentary screen time. We observed that the effectiveness of parental practices for restricting children’s screen time was influenced by the level of family communication. Specifically, we found that when parents restricted their children’s screen time and maintained high levels of family communication, children spent less on recreational sedentary screen time. Our findings also found that while parents’ screen time was significantly associated with more screen time for children, those from families with higher levels of communication might have less screen time even when their parents had more screen time. These results regarding restriction practices and parental screen behavior demonstrated the important role of a positive family communication environment in amplifying the positive effects of screen time limitations and buffering the negative impact of parents’ screen behaviors. This underscores the importance of fostering a healthy communication atmosphere within families. However, positive reinforcement from parents, such as praise for reduced screen time, was surprisingly associated with more recreational screen time among children in families with high communication levels than among those in families with lower communication levels. This unexpected outcome might partly stem from the limitations of our study’s cross-sectional design and possibly be due to children interpreting the positive reinforcement as leniency or mixed messages about screen time norms. Longitudinal studies are needed to clarify the dynamics of parental reinforcement and family communication and their impact on children’s screen behaviors. Overall, our findings suggest the importance of considering multiple family factors and communication dynamics when designing relevant interventions to manage children’s screen time.

Our bivariate analyses also revealed the relationships between demographic factors and recreational sedentary screen time among Chinese primary school-aged children, revealing additional insights. Specifically, our outcome variable significantly varied by sex, age, region of residence, education level of both parents, household income, and family size, indicating the necessity of considering demographic diversities for implications for relevant interventions and strategies to manage screen time among children. Additionally, we also found that children who communicated primarily with grandparents had significantly more recreational sedentary screen time than did those with their parents, highlighting the impact of multigenerational households on children’s behaviors. Recent research revealed that living with grandparents was related to Latino children’s greater odds of having excessive screen time [60]. In addition, existing studies on various populations have emphasized that coresidence with grandparents was associated with children’s dietary habits, BMI, and weight status [60, 61, 62]. Thus, it is crucial to incorporate the role of multigenerational family types in studying childhood obesity and family-based interventions for reducing sedentary screen time in minors.

This study has several limitations. First, due to the cross-sectional nature of our data, we cannot conclude our findings with any causal inferences. Second, all the questionnaires were self-reported, and the participants were minors. Self-reporting bias could exist due to the design and study population of this research, and the outcome variable, children’s daily recreational screen time, could be inaccurate and cause recall bias. In addition, our questionnaire did not include other important family factors, such as parents’ marital status and mental health status, which have been shown to be significantly related to children’s screening time [63, 64]. In addition to our demographic adjustment variables, future studies could consider adjusting for these related family-level factors. Another important limitation is that our questionnaire did not capture sedentary screen time for different digital devices. One study using data across 30 countries revealed that from 2002 to 2010, while children’s screening time for televisions decreased, the average time was still more than 2 h daily for 11-year-olds, and their computer time improved approximately two-fold for both genders [65]. While our current study can serve as an insightful starting point for investigating the relationship between family factors and leisure-related screen time, future studies may focus on the trend of Chinese children’s screen time among different digital devices. In addition, we included only fifth-grade primary school students. Thus, there could be external validity concerns if the results are used to apply to children pre-schooled or those in different grades. Despite these limitations, our study provides important groundwork for understanding the complex relationship between family factors and children’s recreational sedentary screen time. Future studies will be needed to address these concerns, helping to develop more targeted and effective relevant interventions and policies.

## Conclusion

Our study assessed and investigated the recreational sedentary screen time of Chinese primary school-aged children and explored its association with various family factors. The findings underscore the significance of family dynamics, parental practices, and communication in shaping children’s screen-time behaviors, providing valuable insights for tailored interventions and strategies to reduce childhood obesity in the post-COVID era.

### Electronic supplementary material

Below is the link to the electronic supplementary material.

Supplementary Table S1. Multicollinearity results: variance inflation (VIF) by variables.
Supplementary Table S2. Multivariate regression results, coefficients of Model 1–4.
Supplementary Table S3. Multivariate regression results, coefficients of Model 5.

## Data Availability

The datasets used during the current study are available from the corresponding author upon reasonable request.
