# Supplementary material for: The impact of family factors and communication on recreational sedentary screen time among primary school-aged children: a cross-sectional study
Source: BMC Public Health. 2024 Jun 28;24:1733. doi: 10.1186/s12889-024-19128-y (PMC11214221; doi:10.1186/s12889-024-19128-y)
Supplement: Supplementary file 1 — Supplementary Table S1. Multicollinearity results: variance inflation (VIF) by variables. Supplementary Table S2. Multivariate regression results, coefficients of Model 1–4. Supplementary Table S3. Multivariate regression results, coefficients of Model 5. [file 12889_2024_19128_MOESM1_ESM.pdf]

Supplementary Data

Supplementary Table S1. Multicollinearity results: variance inflation (VIF) by variables

Variables	Variance Inflation
Physical environment: Number of devices at home	1.2169
Physical environment: Proportion of Child-owned Devices	1.0512
Physical environment: Child has own room	1.1246
Parental practice: Restrict the Child's Screen Time	1.0507
Parental practice: Less screen time gets praise	1.1156
Parental practice: Family members have screen time together	1.0195
Parent related behavior: Recreational screen sedentary time	1.0534
Parent attitude: Too long sitting time can cause obesity	1.3671
Parent attitude: Too Long Sitting Time Is Bad for Health	1.3783
Parent attitude: I Prefer Sitting than Exercising	1.0413
Parent attitude: I Will Try to Reduce My Sitting Time	1.0648
Family communication quartile	1.2354
Gender	1.0347
Age of Child	1.5752
Minority	1.0182
Region: Uban/Suburban	1.7683
Number of Children in the Household	1.1673
Main Family Member Communicated with the Child Most	1.0828
Father's Education	2.5360
Mother's Education	2.5466
Household Monthly Income	1.4500
Child Schoolwork Related Sitting Time	1.0240
Number of Days per Week: Child with 30+ Minutes Exercising	1.0947

Supplementary Table S2. Multivariate regression results, coefficients of Model 1-4

Variables	Coefficients	P-value
**Model 1: Unadjusted**		
Physical environment: Number of devices at home	0.1327	0.9167
Physical environment: Proportion of Child-owned Devices	-0.0013	0.0005
Physical environment: Child has own room	0.0970	0.0002
**Model 1: Adjusted**		
Physical environment: Number of devices at home	0.0361	0.0146
Physical environment: Proportion of Child-owned Devices	0.1168	0.0041
Physical environment: Child has own room	0.0702	0.0148
**Model 2: Unadjusted**		
Parental practice: Restrict the Child's Screen Time	-0.1214	<.0001
Parental practice: Less screen time gets praise	-0.0641	0.0017
Parental practice: Family members have screen time together	0.0986	0.0002
**Model 2: Adjusted**		
Parental practice: Restrict the Child's Screen Time	-0.1120	<.0001
Parental practice: Less screen time gets praise	-0.0542	0.0137
Parental practice: Family members have screen time together	0.0861	0.0026
**Model 3: Unadjusted**		
Parent related behavior: Recreational screen sedentary time	0.0236	<.0001
Parent attitude: Too long sitting time can cause obesity	0.0149	0.5905
Parent attitude: Too Long Sitting Time Is Bad for Health	-0.0510	0.1118
Parent attitude: I Prefer Sitting than Exercising	-0.0102	0.7311
Parent attitude: I Will Try to Reduce My Sitting Time	-0.0654	0.0043
**Model 3: Adjusted**		
Parent related behavior: Recreational screen sedentary time	0.0207	0.0004
Parent attitude: Too long sitting time can cause obesity	0.0282	0.3458
Parent attitude: Too Long Sitting Time Is Bad for Health	-0.0305	0.3756
Parent attitude: I Prefer Sitting than Exercising	-0.0041	0.8958
Parent attitude: I Will Try to Reduce My Sitting Time	-0.0715	0.0037
**Model 4: Unadjusted**		
Physical environment: Number of devices at home	-0.0036	0.7834
Physical environment: Proportion of Child-owned Devices	0.1135	0.0039
Physical environment: Child has own room	0.0881	0.0009
Parental practice: Restrict the Child's Screen Time	-0.1178	<.0001
Parental practice: Less screen time gets praise	-0.0485	0.0233
Parental practice: Family members have screen time together	0.1031	0.0002
Parent related behavior: Recreational screen sedentary time	0.0206	0.0001
Parent attitude: Too long sitting time can cause obesity	0.0141	0.6162
Parent attitude: Too Long Sitting Time Is Bad for Health	-0.0452	0.1657

Parent attitude: I Prefer Sitting than Exercising	-0.0042	0.8884
Parent attitude: I Will Try to Reduce My Sitting Time	-0.0596	0.0099
**Model 4: Adjusted**		
Physical environment: Number of devices at home	0.0284	0.0639
Physical environment: Proportion of Child-owned Devices	0.1109	0.0088
Physical environment: Child has own room	0.0644	0.0289
Parental practice: Restrict the Child's Screen Time	-0.1103	<.0001
Parental practice: Less screen time gets praise	-0.0378	0.0990
Parental practice: Family members have screen time together	0.0800	0.0074
Parent related behavior: Recreational screen sedentary time	0.0172	0.0036
Parent attitude: Too long sitting time can cause obesity	0.0269	0.3732
Parent attitude: Too Long Sitting Time Is Bad for Health	-0.0277	0.4298
Parent attitude: I Prefer Sitting than Exercising	0.0034	0.9155
Parent attitude: I Will Try to Reduce My Sitting Time	-0.0678	0.0065

Supplementary Table S3. Multivariate regression results, coefficients of Model 5

Variables	Coefficients	P-value
**Model 5: Unadjusted**		
Physical environment: Number of devices at home	0.0247	0.3878
Physical environment: Proportion of Child-owned Devices	0.0163	0.8416
Physical environment: Child has own room	0.0466	0.4622
Number of devices at home * Family communication (2nd vs. 1st quartile)	-0.0082	0.8368
Number of devices at home * Family communication (3rd vs. 1st quartile)	-0.0417	0.2986
Number of devices at home * Family communication (4th vs. 1st quartile)	-0.0646	0.1080
Proportion of Child-owned Devices * Family communication (2nd vs. 1st quartile)	0.0932	0.4188
Proportion of Child-owned Devices * Family communication (3rd vs. 1st quartile)	0.1113	0.3564
Proportion of Child-owned Devices * Family communication (3rd vs. 1st quartile)	0.1160	0.3169
Child has own room * Family communication (2nd vs. 1st quartile)	-0.0099	0.9075
Child has own room * Family communication (3rd vs. 1st quartile)	0.0268	0.7456
Child has own room * Family communication (4th vs. 1st quartile)	0.0853	0.2970
Parental practice: Restrict the Child's Screen Time	0.0351	0.5849
Parental practice: Less screen time gets praise	-0.1045	0.0203
Parental practice: Family members have screen time together	0.0786	0.1628
Restrict the Child's Screen Time * Family communication (2nd vs. 1st quartile)	-0.1448	0.0815
Restrict the Child's Screen Time * Family communication (3rd vs. 1st quartile)	-0.1166	0.1558
Restrict the Child's Screen Time * Family communication (4th vs. 1st quartile)	-0.2822	0.0005
Less screen time gets praise * Family communication (2nd vs. 1st quartile)	0.0750	0.2317
Less screen time gets praise * Family communication (3rd vs. 1st quartile)	0.0355	0.5845
Less screen time gets praise * Family communication (4th vs. 1st quartile)	0.2072	0.0038
Family members have screen time together * Family communication (2nd vs. 1st quartile)	-0.0369	0.6431
Family members have screen time together * Family communication (3rd vs. 1st quartile)	0.0215	0.7902
Family members have screen time together * Family communication (4th vs. 1st quartile)	0.0600	0.4923
Parent related behavior: Recreational screen sedentary time	0.0567	<.0001
Parent attitude: Too long sitting time can cause obesity	0.0959	0.1157
Parent attitude: Too Long Sitting Time Is Bad for Health	-0.1135	0.1028
Parent attitude: I Prefer Sitting than Exercising	-0.0837	0.2173
Parent attitude: I Will Try to Reduce My Sitting Time	-0.0434	0.3985
Recreational screen sedentary time * Family communication (2nd vs. 1st quartile)	-0.0243	0.1439
Recreational screen sedentary time * Family communication (3rd vs. 1st quartile)	-0.0493	0.0042
Recreational screen sedentary time * Family communication (4th vs. 1st quartile)	-0.0605	0.0005
Too long sitting time can cause obesity * Family communication (2nd vs. 1st quartile)	-0.0673	0.4240
Too long sitting time can cause obesity * Family communication (3rd vs. 1st quartile)	-0.1023	0.2264
Too long sitting time can cause obesity * Family communication (4th vs. 1st quartile)	-0.1325	0.1284
**Model 5: Adjusted**		
Physical environment: Number of devices at home	0.0584	0.0429

Physical environment: Proportion of Child-owned Devices	0.0590	0.4769
Physical environment: Child has own room	-0.0173	0.7862
Number of devices at home * Family communication (2nd vs. 1st quartile)	-0.0011	0.9774
Number of devices at home * Family communication (3rd vs. 1st quartile)	-0.0504	0.2105
Number of devices at home * Family communication (4th vs. 1st quartile)	-0.0670	0.0955
Proportion of Child-owned Devices * Family communication (2nd vs. 1st quartile)	0.0517	0.6562
Proportion of Child-owned Devices * Family communication (3rd vs. 1st quartile)	0.0757	0.5360
Proportion of Child-owned Devices * Family communication (3rd vs. 1st quartile)	0.0434	0.7119
Child has own room * Family communication (2nd vs. 1st quartile)	0.0326	0.7047
Child has own room * Family communication (3rd vs. 1st quartile)	0.1052	0.2079
Child has own room * Family communication (4th vs. 1st quartile)	0.1357	0.1007
Parental practice: Restrict the Child's Screen Time	0.0324	0.6167
Parental practice: Less screen time gets praise	-0.0853	0.0592
Parental practice: Family members have screen time together	0.0509	0.3696
Restrict the Child's Screen Time * Family communication (2nd vs. 1st quartile)	-0.1279	0.1268
Restrict the Child's Screen Time * Family communication (3rd vs. 1st quartile)	-0.1092	0.1896
Restrict the Child's Screen Time * Family communication (4th vs. 1st quartile)	-0.2774	0.0007
Less screen time gets praise * Family communication (2nd vs. 1st quartile)	0.0517	0.4106
Less screen time gets praise * Family communication (3rd vs. 1st quartile)	0.0288	0.6602
Less screen time gets praise * Family communication (4th vs. 1st quartile)	0.1749	0.0152
Family members have screen time together * Family communication (2nd vs. 1st quartile)	-0.0091	0.9099
Family members have screen time together * Family communication (3rd vs. 1st quartile)	0.0240	0.7729
Family members have screen time together * Family communication (4th vs. 1st quartile)	0.0896	0.3065
Parent related behavior: Recreational screen sedentary time	0.0526	<.0001
Parent attitude: Too long sitting time can cause obesity	0.0948	0.1219
Parent attitude: Too Long Sitting Time Is Bad for Health	-0.0941	0.1771
Parent attitude: I Prefer Sitting than Exercising	-0.0789	0.2513
Parent attitude: I Will Try to Reduce My Sitting Time	-0.0438	0.3988
Recreational screen sedentary time * Family communication (2nd vs. 1st quartile)	-0.0244	0.1519
Recreational screen sedentary time * Family communication (3rd vs. 1st quartile)	-0.0539	0.0024
Recreational screen sedentary time * Family communication (4th vs. 1st quartile)	-0.0590	0.0009
Too long sitting time can cause obesity * Family communication (2nd vs. 1st quartile)	-0.0559	0.5093
Too long sitting time can cause obesity * Family communication (3rd vs. 1st quartile)	-0.0868	0.3106
Too long sitting time can cause obesity * Family communication (4th vs. 1st quartile)	-0.1328	0.1314
